# Emergence of *Cryptosporidium parvum* IIc Subtype and *Giardia duodenalis* Assemblage E in AIDS Patients in Central China: Evidence for Neglected Transmission Dynamics

**DOI:** 10.3390/microorganisms13081731

**Published:** 2025-07-24

**Authors:** Zhuolin Tao, Ke Hong, Peixi Qin, Hui Liu, Chunqun Wang, Jigang Yin, Xin Li, Guan Zhu, Min Hu

**Affiliations:** 1 National Key Laboratory of Agricultural Microbiology, College of Veterinary Medicine, Huazhong Agricultural University, Wuhan 430070, China; taozhuolin@webmail.hzau.edu.cn (Z.T.); peixiqin@webmail.hzau.edu.cn (P.Q.); liuhui45@webmail.hzau.edu.cn (H.L.); wangchunqun@mail.hzau.edu.cn (C.W.); 2 Wuhan Jinyintan Hospital, Tongji Medical College of Huazhong University of Science and Technology, Hubei Clinical Research Center for Infectious Diseases, Wuhan Research Center for Communicable Disease Diagnosis and Treatment, Chinese Academy of Medical Sciences, Joint Laboratory of Infectious Diseases and Health, Wuhan Institute of Virology and Wuhan Jinyintan Hospital, Chinese Academy of Sciences, Wuhan 430023, China; 38086802@163.com; 3 State Key Laboratory for Diagnosis and Treatment of Severe Zoonotic Infectious Diseases, Key Laboratory for Zoonosis Research of the Ministry of Education, Institute of Zoonosis, College of Veterinary Medicine, Jilin University, Changchun 130062, China; yinjg@jlu.edu.cn (J.Y.); lixin2018@jlu.edu.cn (X.L.); zhuguan@jlu.edu.cn (G.Z.)

**Keywords:** HIV/AIDS, *Cryptosporidium*, *Giardia duodenalis*, *Enterocytozoon bieneusi*, molecular characterization, zoonotic transmission, cross-border transmission

## Abstract

Zoonotic opportunistic enteric protozoa represent a significant global health threat to immunocompromised populations, especially individuals with human immunodeficiency virus (HIV). Despite China’s severe HIV burden, molecular epidemiological data on enteric protozoa remain limited in this population. In this study, we investigated the occurrence and molecular characteristics of *Cryptosporidium* species, *Giardia duodenalis*, and *Enterocytozoon bieneusi* among 150 AIDS patients with severe immunodeficiency in Wuhan city, Hubei Province, China. The overall test-positive rate was 5.33% (8/150), comprising *Cryptosporidium* species (including *C. hominis*, *C. parvum,* and *C. meleagridis*) in 2.00% (3/150) and *G. duodenalis* (including assemblage A, B, and E) in 3.33% (5/150); *E. bieneusi* was not detected. Notably, this study reports the first identification of the *C. parvum* subtype IIcA5G3 in humans in China, certainly indicating possible cross-border transmission. Furthermore, the detection of *C. meleagridis* IIIbA22G1R1c provided additional molecular evidence for chicken-to-human transmission. The finding of *G. duodenalis* assemblage E highlights the underrecognized zoonotic spillover risks to immunocompromised populations. These findings emphasize the diversity of infectious reservoirs, and the need for enhanced national molecular surveillance of these neglected zoonotic enteric protozoa, alongside targeted interventions for vulnerable populations.

## 1. Introduction

Acquired immunodeficiency syndrome (AIDS) caused by human immunodeficiency virus (HIV) has been one of the most devastating persisting pandemics of this era [1]. To date, there are 39.9 million people living with HIV (PLHIV) worldwide [2], and the progressive depletion of the cluster of differentiation 4 positive (CD4+ T) lymphocytes in PLHIV establishes an immunocompromised milieu conducive to opportunistic infections across bacterial, fungal, viral, and parasitic taxa, significantly elevating the risks of life-threatening complications [3,4,5,6,7,8].

Among parasitic protists, three zoonotic enteric protozoa demand heightened attention in immunocompromised populations: at least 19 species and 4 genotypes of *Cryptosporidium*, 6 assemblages (A–F) of *Giardia duodenalis*, and 106 genotypes of *Enterocytozoon bieneusi* have been reported in humans [9,10,11]. These pathogens exploit fecal–oral transmission routes via anthroponotic, zoonotic, or environmental pathways [12,13]. Infections exhibit a wide spectrum of clinical manifestations and vary among each pathogen. Common symptom clusters encompass diarrhea, malabsorption, wasting, etc., which could be moderate to severe, but are generally self-limiting or even asymptomatic in immunocompetent individuals [14]. Although antiretroviral therapy (ART) can restore immunity to these pathogens in HIV-1-infected individuals, eradicating the infection could still be a great challenge [15]. In addition, drug therapy for these enteric protozoa is far from optimal for PLHIV [16,17,18], which increases the risks of these infections to this population, exhibiting a considerable and increasing global burden, especially in developing countries with poverty [19].

Despite China’s severe HIV burden (1.329 million existing cases) [20], molecular epidemiological data on enteric protozoa in PLHIV remain strikingly limited. To date, only five epidemiological studies in China have characterized these pathogens at the molecular level among the PLHIV in Henan [21], Guangxi [22], Shanghai [23], Heilongjiang [24], and Jiangxi [25], respectively. Therefore, the actual situation remains far from fully elucidated, with critical knowledge gaps persisting concerning both regional heterogeneity in pathogen diversity and dominant species/genotypes and the respective contributions of anthroponotic vs. zoonotic transmission pathways.

Wuhan, as the largest city in Central China and one of the pivotal hubs in China’s railway, highway, shipping, and aviation networks, with large-scale population flow [26], exemplifies high-risk transmission interfaces between local and migrant populations, evidenced by its critical role in the early national dissemination of SARS-CoV-2 [27,28]. Previous studies in Hubei identified the enteric protozoa mentioned above in children, animals, and wastewater [29,30,31,32,33], while the associated data of PLHIV remain absent. Therefore, we investigate the incidence and molecular characteristics of *Cryptosporidium* spp., *G. duodenalis,* and *E. bieneusi* in AIDS patients, attempting to provide valuable information for a more in-depth understanding of the epidemiology and to seek evidence for potential sources of infection.

## 2. Materials and Methods

### 2.1. Collection of Specimens and Information

This study was conducted at Wuhan Jinyintan Hospital, in China, which is a designated medical institution for HIV/AIDS patients and virus carriers in Hubei Province, from September 2023 to April 2025. Eligible participants met the following inclusion criteria: (i) a confirmed HIV infection through nucleic acid amplification testing (NAAT) of viral RNA, and (ii) severe immunodeficiency defined as a CD4+ T-cell count < 200 cells/μL, measured within 48 h prior to enrollment.

Demographic and clinical data were systematically collected using a structured questionnaire. Key variables included age, sex, residential address (categorized as urban or rural), and most recent CD4+ T-cell count. Clinical manifestations of interest encompassed gastrointestinal symptoms (diarrhea, abdominal pain, vomiting, decreased appetite) and systemic features (fever, fatigue). Additional data on diagnosed co-infections (e.g., *Peumocystis carinii* peumonia, tuberculosis) were recorded when available.

Fresh stool specimens provided by the participants were immediately preserved by mixing with an equal volume of 2.5% (*w*/*v*) potassium dichromate solution and stored at 4 °C for subsequent molecular analysis.

### 2.2. Genomic DNA Extraction

FastDNA^TM^ SPIN Kit for Feces (MP Biomedical, Santa Ana, CA, USA) was employed to extract genomic DNA. A total of 0.5 mL of fecal materials was pipetted into a Matrix E tube, washed, and centrifuged twice with phosphate-buffered saline (PBS) solution; then, genomic DNA was extracted following the manufacturer-recommended procedures and stored at −20 °C until use.

### 2.3. Nested PCR

Nested PCR assays targeting the specific genes of each pathogen were conducted as follows: For *Cryptosporidium* spp., small subunit ribosomal RNA (SSU rRNA) was employed for species identification and classification [34], and the positive samples were further subtyped by sequence analyses of the 60-kDa glycoprotein gene (*gp60*) following established protocols [35,36]. For *G. duodenalis*, the triosephosphate isomerase (*tpi*), β-giardin (*bg*), and the glutamate dehydrogenase (*gdh*) genes were employed, and genetic assignment was to the level of assemblage [37,38]. For *E. bieneuci*, the internal transcribed spacer (ITS) of the nuclear ribosomal RNA gene was employed [39]. 2 × SanTaq PCR Mix (Sangong Biotech, Shanghai, China) was utilized for all reactions, and thermal cycling conditions followed the published protocols for each primer set (Table S1). Known test-positive genomic DNA (*C. parvum* IIdA19G1 from a calf, *G. duodenalis* assemblage E from cattle, *G. duodenalis* assemblage B from a rabbit, and *E. bieneusi* genotype 1 from cattle) and no-template controls (reagent-grade water) were included in each set of PCRs to ensure the absence of cross-contamination. Amplicons were visualized on 1.5% agarose gels stained with a Super Red nucleic acid stain (Biosharp life science, Hefei, China).

### 2.4. Indirect Immunofluorescence Microscopy Assay

Indirect immunofluorescence microscopy was performed on the positive specimens from the nested PCR for further verification. After preliminary purification to remove coarse debris, sediment of the 500 μL resuspended specimens was blocked with a 3% bovine serum albumin (BSA) in PBS (37 °C, 30 min) in 1.5 mL centrifuge tubes, then sequentially incubated with a primary antibody (1:500 dilution in 1% BSA/PBS; 4 °C, 12 h) and a fluorophore-conjugated secondary antibody (1:1000 dilution in PBS; 37 °C, 1 h). All incubations were followed by three PBS wash cycles (10,000× *g*, 5 min), and the samples were resuspended with 200 μL of PBS. *Cryptosporidium* oocysts were examined using a chicken yolk antibody against total *C. parvum* oocyst proteins paired with Alexa Fluor^®^ 488-conjugated goat anti-chicken IgG (Abcam, Cambridge, UK). *G. duodenalis* cysts were examined using a mouse polyclonal antibody against prokaryotic recombinant β-giardin protein and CoraLite^®^ 488-conjugated goat anti-mouse IgG (Proteintech, Wuhan, China). Three technical replicates per sample were examined by fluorescence microscopy under 488 nm excitation, with 10 μL of the final suspension mounted on glass slides.

### 2.5. Sequencing and Nucleotide Sequence Analysis

The PCR amplicons from the secondary PCR program of expected length were purified, and then bi-directionally sequenced on an ABI 3730Xl (Applied Biosystems, Foster City, CA, USA) by Tsingke Biotechnology (Beijing, China). The obtained nucleotide sequences were assembled using ChromasPro2.1.6 (http://technelysium.com.au/ChromasPro.html (accessed on 19 July 2025)), then aligned with each other and reference sequences which were downloaded from GenBank with their corresponding accession numbers (accession nos.) (https://www.ncbi.nlm.nih.gov (accessed on 19 July 2025)) using ClustalX 2.1 (www.clustal.org (accessed on 19 July 2025)).

### 2.6. Statistical Analysis

Given the low expected frequencies (<5 counts in >20% of cells) observed, categorical data analysis was performed using Fisher’s exact test (for 2 × 2 comparisons) or the Fisher-Freeman–Halton test (for r × c tables, r > 2 or c > 2) in SPSS software 26.0 (IBM Corp., Armonk, NY, USA). Statistical significance was defined as a two-tailed *p*-value < 0.05.

## 3. Results

### 3.1. Demographic Information

**Figure 1:**
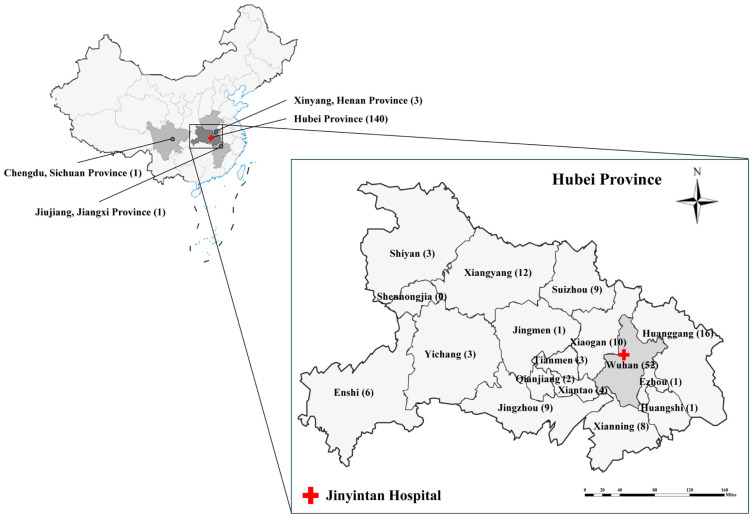
Geographical distribution of patient origins and the location of Jinyintan Hospital. Among 150 patients, 140 were from 13 cities in Hubei Province, with 5 additional cases from three adjacent provincial cities (Xinyang, Henan Province; Jiujiang, Jiangxi Province; Chengdu, Sichuan Province). Geographical information was unavailable for five patients.

During the study period, fecal specimens were collected from 150 AIDS participants, among whom 140 were from 13 cities or areas in Hubei province, with 5 residing in 3 nearby provinces, while the remaining 5 did not provide complete information (Figure 1) (Table S2). The age of the subjects was between 19 and 80 years old (mean age  =  46.5 years old, median age = 48 years old), and the sex ratio (male–female) in the group was 135:11. The ratio of the residential areas of patients (urban–rural) was 57:89. Of the participants, 33.56% (49/146) were receiving ART, whereas 66.44% (97/146) were ART-naive or ART-experienced but discontinued (Table 1).

### 3.2. Occurrence of Cryptosporidium spp., Giardia duodenalis, and Enterocytozoon bieneusi

The overall test-positive rate of the three enteric protozoan infections was 5.33% (8/150). The test positive rate of *Cryptosporidium* spp. was 2.00% (3/150). Three *Cryptosporidium* species, including *C. meleagridis* (1 case), *C. hominis* (1 case), and *C. parvum* (1 case), were detected in three patients and successfully genotyped via a sequence analysis of the SSU rRNA gene (Table 1 and Table 2). *Giardia duodenalis* was detected in five patients, including assemblage A (1 case), assemblage B (3 cases), and assemblage E (1 case), and its test positive rate was 3.33% (5/150) (Table 1 and Table 3). None of the specimens tested positive for *E. bieneusi*, or for co-infection of *Cryptosporidium* spp. and *G. duodenalis.*

**Figure 2:**
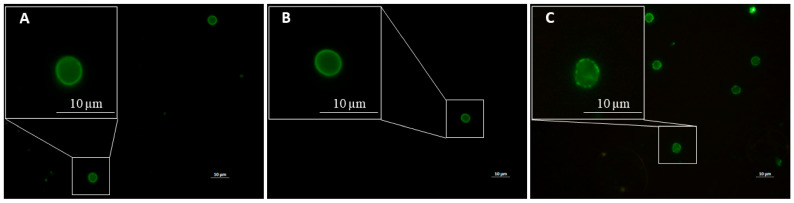
Oocysts of *Cryptosporidium* via indirect immunofluorescence assay. Oocysts of *Cryptosporidium meleagridis* (**A**), *Cryptosporidium hominis* (**B**), and *Cryptosporidium parvum* (**C**) displaying apple-green fluorescence (excitation 488 nm) via indirect immunofluorescence microscopy. Scale bar = 10 μm.

In the indirect immunofluorescence assay, the nested PCR-confirmed *Cryptosporidium* spp.-positive samples (n = 3) consistently exhibited intact oocysts with characteristic apple-green fluorescence under 488 nm excitation, whereas no cyst structures were visualized in any *Giardia duodenalis*-positive specimens, despite confirmed DNA detection by nested PCR (Figure 2). The microscopic examination exhibited inherently lower analytical sensitivity than the molecular assays, potentially accounting for suboptimal detection in the specimens with low fecal loads [40,41].

### 3.3. Characterization of Subtypes of Cryptosporidium spp. and Assemblages of Giardia duodenalis

According to the sequencing results of *gp60* locus, the detected *C. hominis*, *C. parvum*, and *C. meleagridis* were further characterized as subtypes IfA12G1R5, IIcA5G3, and IIIbA22G1R1c, respectively (Table 2). Two of the five *G. duodenalis*-positive specimens were successfully amplified and genotyped at *tpi*, *bg,* and *gdh* loci (No. 1 and No. 2), and both were classified as assemblage B. The remaining three specimens were amplified and genotyped at only one locus, two at *bg* (No. 3 and No. 4) and one at *tpi* (No. 5) (Table 3).

All sequences obtained in this study demonstrated 100% nucleotide identity to the reference sequences deposited in GenBank, with host-geographical patterns across pathogen species or assemblages. The sequence of *C. hominis* showed 100% identity with reference sequences derived from several developed nations, while the sequence of *C. parvum* subtype IIc demonstrated exclusive phylogenetic clustering with clinical specimens collected from HIV-infected patients in Africa (Accession nos. AF440621, EU141721, and AF440631), which was consistent with the patient’s self-reported occupational exposure in Africa. Additionally, the sequence of *gp60* from *C. meleagridis* showed genomic congruence with clinical isolates obtained from children with diarrhea in Hubei Province (Accession no. KY575457), with additional matches to poultry-derived strains in the same region (Accession no. MG969391) (Table 2).

For *G. duodenalis* assemblage B, the sequences of *bg* locus revealed divergent host specificity. Two sequences exclusively matched human-derived references (100% identity), while a third specimen (PV683348) additionally shared identity with a non-human primate (hylobatidae) isolate from China (KY696833). The sequences of *tpi* and *gdh* loci were exclusively conserved, and the sequences of *gdh* loci showed 100% identity to that derived from a non-human primate (hylobatidae) and a rabbit in China. For assemblage A, the *tpi* locus sequence matched with the sequences of a broad range of host species (Table S3). For assemblage E, the *bg* sequence differed from the positive controls and all prior isolates of our laboratory. However, it clustered with and exhibited 100% identity to a cattle-derived isolate (KY769091) from Chengdu, the patient’s residential area, thereby suggesting local livestock adaptation (Table 3). Collectively, these findings delineated distinct potential transmission patterns spanning anthroponotic spread (*C. parvum* IIc subtype), broad-spectrum zoonotic reservoirs (encompassing *C. meleagridis* and *G. duodenalis* including assemblages A and B), and potential livestock-adapted cycles (*G. duodenalis* assemblage E).

### 3.4. Statistical Analyses of Cryptosporidium spp. and Giardia duodenalis Infection Risks

The initial analysis using Fisher’s exact test demonstrated no significant associations between the infections of individual protozoan (*Cryptosporidium* spp. or *G. duodenalis*) and demographic stratification by age, gender, or residential area (*p* > 0.05). However, upon a group-wise amalgamation of the infection events, Fisher’s exact test revealed that elevated infection prevalence among rural populations showed statistically significant associations (*p* = 0.016) (Table 1).

Clinical profiling identified fatigue as the predominant symptom (29.45%, 43/146), succeeded by diarrhea (18.49%, 27/146), fever (17.12%, 25/146), vomiting (16.44%, 24/146), appetite loss (14.38%, 21/146), abdominal pain (6.16%, 9/146), and nausea (5.48%, 8/146). Only diarrhea exhibited statistically significant associations through Fisher’s exact tests, linking it to both *Cryptosporidium* infection (*p* = 0.005) and cumulative cases of *Cryptosporidium* and *G. duodenalis* infections (*p* = 0.046). All *Cryptosporidium*-positive cases clustered exclusively within the diarrheal subgroup (3/27 vs. 0/119-non-diarrheal cases), and the cumulative incidence of *Cryptosporidium* spp./*G. duodenalis* mono-infections was six-fold higher in diarrhea patients (14.81% vs. 2.52% in non-diarrheal cases) (Table 4). Detailed information on the test-positive patients is listed in Table S4.

As all samples were categorized by their collection seasons, *Cryptosporidium* spp. was detected only in specimens collected in the spring and winter, with positivity rates of 6.25% (2/32) and 1.89% (1/53), respectively. For *G. duodenalis*, the rates were 9.38% (3/32) in the spring vs. 3.77% (2/53) in the winter. None of the specimens collected in the summer (n = 36) or the autumn (n = 29) tested positive. Initial analyses using the Fisher–Freeman–Halton test (for 2 × 4 contingency tables) revealed no significant seasonal trends for either pathogen individually (*Cryptosporidium* spp.: *p* = 0.394; *G. duodenalis*: *p* = 0.221) or the pooled cases (*p* = 0.102) (Table 5).

## 4. Discussion

HIV/AIDS patients may function as “sentinel” populations for epidemiological surveillance in the context of immunocompromised host–pathogen interactions. Their distinct spectrum of opportunistic infections provides critical insights into identifying geographically restricted, emerging, or neglected pathogens [42]. Here, we identified *C. hominis* subtype IfA12G1R5, *C. parvum* subtype IIcA5G3, *C. meleagridis* subtype ⅢbA22G1R1, as well as *G. duodenalis* assemblages A, B, and E in AIDS patients, and formulated reliable hypotheses of different transmission routes or sources of infection.

In this study, we reported the first IIcA5G3 subtype of *C. parvum*, a subtype previously unreported in clinical surveillance in China, which is an almost exclusively anthroponotic subtype family, also designated *C. parvum anthroponosum* [9,43,44]. The IIc subtype causes the predominant *C. parvum* infection in low- and middle-income countries globally, especially in HIV-positive populations [45,46]. According to the patient’s self-reported occupational exposure in Africa, which is a recognized prevalent region for the IIc subtype, aligns with the hypothesis of cross-border transmission. Crucially, this case marks the first confirmed human infection with the IIc subtype within China, challenging its prior epidemiological absence. However, critical knowledge gaps persist regarding local transmission risks: the indeterminate infection timeline and incomplete activity tracing preclude a robust assessment of potential secondary transmission chains within the patient’s residential community. Worth mentioning is the import of *C. parvum* IIa subtypes into China, which is one of the main causes of human cryptosporidiosis in industrialized nations, and has occurred among grazing ruminants, posing insufficiently evaluated risks to both human and ruminant health domestically [47]. In light of this, the identification of the IIc subtype further underscores the necessity of enhanced molecular surveillance, which is critical to characterize transmission dynamics and evaluate clinical implications, particularly among immunocompromised and other vulnerable populations. The patient infected with *C. meleagridis* further enhances the current understanding of *C. meleagridis* transmission dynamics in Hubei province, with a confirmed 100% identity at the *gp60* locus between the chicken-derived isolate [32], and the human-derived isolates including the AIDS patient and children [30]. The finding constitutes compelling phylogenetic evidence supporting zoonotic transmission, while the potential anthroponotic transmission mentioned previously cannot be discounted or excluded [46], particularly among children and immunodeficient populations.

Additionally, we also identified the IfA12G1R5 subtype of *C. hominis*, which is a relatively infrequent subtype in China. While this subtype has been recurrently reported in industrialized countries [48,49,50,51,52], one recent study has revealed that the predominant *C. hominis* hypertransmissable subtype IfA12G1R5 in the United States emerged from multiple introductions and recombination events, including IfA12G1R5 originating from Europe [53]. This finding underscores the importance of paying close attention to this particular subtype. The first documentation of IfA12G1 in China occurred only recently in Xinjiang and this subtype appears to maintain a sporadic pattern domestically [54]. The significant geographical separation between Xinjiang and Hubei (>2300 km apart) likely precludes direct human-to-human transmission chains between these regions. Nevertheless, potential transmission vectors involving transient population mobility or contaminated agricultural supply chains cannot be conclusively ruled out. However, it is a pity that the sequence data are not available for further comparing these two sequences. The If R repeat is complicated, and had been defined differently resulting in an alternative subtype nomenclature. It was not until 2025 that the R repeat designation was clarified [55]. Therefore, whether the sequence of IfA12G1 isolate contains R repeat sequence was unclear. Another plausible explanation might be that this subtype has exhibited sporadic transmission in at least two geographically distinct regions within China. One situation that requires greater concern might be that this subtype entered China through cross-border transmission, whereas the participant denied any overseas experience, and the absence of regional epidemiological data precludes further investigation for tracing. Whole genome sequencing (WGS) on the specimen in the future might validate our speculation. We should be more vigilant to avoid following in the footsteps of the United States, especially in the context of increasing population mobility, and call for integrating transnational health surveillance into national HIV programs. It is worth noting that at least two other studies documented different subtypes of the If family in China—among rhesus monkeys (*Macaca mulatta*) and in Shanghai’s wastewater systems [36,56]. Given the vast geographical heterogeneity of the *C. hominis* subtypes across China, current surveillance data remain critically insufficient. Complementary investigations targeting multisource objects (human, environmental, and zoonotic reservoirs) are imperative to elucidate the transmission networks of this protozoan.

For *G. duodenalis*, assemblage E is generally considered a hoofed livestock-specific genotype, while previous studies have broadened its host ranges in animals such as rabbits and non-human primates [57,58], and it has also been reported globally in over 50 human giardiasis cases in recent years [12]. In contrast, assemblage A has a broad range of mammalian hosts, especially subtype AI [59]. Notably, the detection of *G. duodenalis* assemblage E in the patient from Chengdu, Sichuan Province in our study, coupled with its 100% identity at *bg* locus to calf-derived sequences from Chengdu [60]. This finding suggests a potential zoonotic transmission of assemblage E in the region, mirroring the potential zoonotic patterns observed in other areas, such as Egypt and Australia [40,61]. In recent research, phylogenetic analysis supports the zoonotic transmission of *G. intestinalis* assemblage E from bovine to human hosts in agricultural settings in Egypt [62]. In addition, it is considered that assemblage E also presents both zoonotic and anthroponotic profiles [63]. These findings underscore the importance of enhancing surveillance systems for zoonotic protozoan parasites among both human and animal populations in rural and underdeveloped regions, in order to implement targeted intervention strategies for mitigating transmission risks. However, the limited clinical and epidemiological metadata in this study preclude definitive conclusions regarding transmission dynamics. Therefore, in the “One Health” framework, targeted investigations incorporating molecular surveillance of both human and livestock populations in this region, and genotyping based on other gene loci or WGS are warranted to validate these hypotheses.

Despite robust evidence for local transmission patterns, certain limitations should be noted. This study was conducted at a relatively low scale (only 150 patients enrolled), so the test-positivity could not convictively represent the overall prevalence in this population in Central China. Despite prior reported cases of *E. bieneusi* in Wuhan [30], none of the cases tested positive among AIDS patients, which seems to contrast with their heightened susceptibility to opportunistic pathogens. Here are three plausible explanations: First, the limited scale might preclude the finding of infected cases (95% CI: 0–2.5%). Second, while low pathogen loads may evade identification. Nested PCR remains the dominant technique for molecular epidemiology due to its direct genotyping capability via amplicon sequencing. Novel methods with superior sensitivity show future potential for human diagnostics [64,65]. Third, regional epidemiological variation might be neglected, which necessitates integrated surveillance across human, animal, and environmental reservoirs. Furthermore, due to the lack of precise temporal data on infection onset (a common limitation in cross-sectional studies compared to cohort designs), this study could not reliably assess the association between enteric protozoan infections and seasonal variations, which might also be a limitation in previous studies [66]. These limitations highlight the need for future work on (i) larger-scale population enrollment to enhance statistical power, (ii) systematic clinical and exposure-related metadata collection covering demographics, behavioral patterns, and histories of direct or environmental animal contact, and (iii) comparative genome-level analysis of isolates derived from different hosts to gather more comprehensive, directly linked, and more compelling evidence associated with these transmission dynamics.

## 5. Conclusions

This study uncovers the transmission risks of *Cryptosporidium* spp. and *G. duodenalis* among AIDS patients in Central China, holding direct implications for poverty-linked zoonotic infectious disease control. The first reported detection of *C. parvum* subtype IIcA5G3 phylogenetically linked to HIV-infected populations in Africa underscores cross-border pathogen dispersion through mobile populations, emphasizing the need to integrate transnational health surveillance into national HIV programs. Concurrently, the identification of *C. meleagridis* (ⅢbA22G1R1c), and *G. duodenalis* assemblages A and E in AIDS patients highlights zoonotic transmission, the vulnerabilities of rural areas, and the urban–rural interface. These findings might position AIDS patients as “sentinel” populations for monitoring zoonotic opportunistic pathogens and other “One Health” threats, urging us to pre-emptively address neglected or emerging protozoan prevalence.

## Figures and Tables

**Table 1:** Demographic characteristics, and infections of *Cryptosporidium* spp. and *Giardia duodenalis* among enrolled AIDS patients ^1^.

Groups		Number of Cases	*Cryptosporidium* spp.		*G*. *duodenalis*		Combined Mono-Infections	
			Positive [n (%)]	*p*-Value	Positive [n (%)]	*p*-Value	Positive [n (%)]	*p*-Value
Age	19–34	36	0	0.592	0	0.268	0	0.070
	35–49	41	2 (4.88)		3 (7.32)		5 (12.20)	
	50–64	57	1 (1.75)		2 (3.51)		3 (5.26)	
	65–80	12	0		0		0	
Gender	Male	135	2 (1.48)	0.344	5 (3.70)	1.000	6 (4.44)	0.223
	Female	11	1 (9.09)		0		1 (9.09)	
Residential area	Urban	57	0	0.294	0	0.144	0	0.016
	Rural	89	3 (3.37)		5 (5.62)		8 (8.99)	
Receiving ART therapy	Yes	49	0	0.293	3 (6.12)	0.674	3 (6.12)	0.697
	No	97	3 (3.09)		2 (2.06)		5 (5.15)	

^1.^ Only 146 patients provided complete demographic data.

**Table 2:** Sequence analysis of SSU rRNA and *gp60* loci of *Cryptosporidium* spp.

Species (Accession No.)	Subtype (Accession No.)	Representative Sequences with 100% Identity in *gp60* Analysis ^1^		
		Host	Area	Accession No.
*C. hominis* (PV664588)	IfA12G1R5 (PV672204)	human	UK	EU161655
			Southern Ireland	MT053131
			Netherland	MH796380
			Australia	GU933448
*C. parvum* (PV664589)	IIcA5G3 (PV672205)	human	South Africa ^2^	AF440621
			Jamaica ^2^	EU141721
			Mozambique ^2^	AF440631
*C. meleagridis* (PV664587)	IIIbA22G1R1c (PV672203)	human	India	KJ210607
			China	KY575457
		chicken	China	MG969391

^1.^ Accession nos. of sequences with 100% identity are listed in Table S3; ^2^ Sequences were derived from fecal specimens of HIV-infected individuals.

**Table 3:** Sequence analysis of *bg*, *tpi,* and *gdh* loci of *Giardia duodenalis*-positive specimens.

Target Gene	Assemblage (n)	Specimen Number	Accession No.	Sequences with 100% Identity ^1^	
				Host	Accession No.
*bg*	Assemblage B (3)	No. 1	PV683349	human	OP947121
		No. 2	PV683351	human	LC436571
		No. 3	PV683348	human	KP687755
				hylobatidae	KY696833
	Assemblage E (1)	No. 4	PV683347	sheep	MN833266
				calf/cattle	KY769091 ^2^
				horse	MN174850
*tpi*	Assemblage A (1)	No. 5	PV700082	human	OR453274
				cattle	OL694043
				yak	MH230890
				sheep	MN833280
	Assemblage B (2)	No. 1	PV672206	human	LN626348
		No. 2	PV672207		
*gdh*	Assemblage B (2)	No. 1	PV672208	human	EF507682
		No. 2	PV672209	hylobatidae	KY696799
				rabbit	KC960645

^1^ Accession nos. of sequences showing 100% identity in sequence analysis, with host and geographic location arelisted in Table S3. ^2^ Sequence was derived from fecal specimens of cattle in Chengdu, which is the residential area of the patient infected with *G. duodenalis* assemblage E.

**Table 4:** Statistical analysis of infection occurrence with immunity status, therapeutic status, and clinical manifestations^1^.

Clinical Manifestation	Categories	Number of Cases	*Cryptosporidium* spp.		*G. duodenalis*		Combined Mono-Infections	
			Positive [n (%)]	*p*-Value	Positive [n (%)]	*p*-Value	Positive [n (%)]	*p*-Value
CD4+ T cell count	<100	109	3 (2.75)	0.552	3 (2.75)	0.645	6 (5.50)	0.433
	101–200	37	0		2 (5.41)		2 (5.41)	
Diarrhea ^2^	Yes	27	3 (11.11)	0.005	1 (3.70)	0.34	4 (14.81)	0.046
	No	119	0		4 (3.36)		4 (3.36)	
Vomiting	Yes	8	0	1.000	0	1.000	0	0.359
	No	138	3 (2.17)		5 (3.62)		8 (5.80)	
Decreased appetite	Yes	21	1 (4.76)	0.478	0	0.326	1 (4.76)	0.438
	No	125	2 (1.60)		5 (4.00)		7 (5.60)	
Abdominal pain	Yes	9	0	1.00	1 (11.1)	0.340	1 (11.1)	0.435
	No	137	3 (2.19)		4 (2.92)		7 (5.11)	
Fever	Yes	25	1 (4.00)	0.513	0	0.319	1 (4.00)	0.435
	No	121	2 (1.65)		5 (4.13)		7 (5.79)	
Fatigue	Yes	43	0	0.293	3 (6.98)	0.088	3 (6.98)	0.418
	No	103	3 (2.91)		2 (1.94)		5 (4.85)	

^1^ Only 146 patients provided complete demographic data. ^2^ The patients were diagnosed as having diarrhea if their Bristol stool scale value was ≥ 6.

**Table 5:** Number and positivity rates of specimens collected in each season.

Season (Months)	Number of Specimens	*Cryptosporidium* spp.		*Giardia duodenalis*		Total Number of Test-Positive	
		Positive [n (%)]	*p*-Value	Positive [n (%)]	*p*-Value	Positive [n (%)]	*p*-Value
Winter (Dec–Feb)	53	1 (1.89)	0.394	2(3.77)	0.221	3 (5.67)	0.102
Spring (Mar–May)	32	2 (6.25)		3 (9.38)		5 (15.63)	
Summer (Jun–Aug)	36	0		0		0	
Autumn (Sep–Nov)	29	0		0		0	

## Data Availability

Nucleotide sequence data reported in this article were submitted and publicly available in the GenBank database (https://www.ncbi.nlm.nih.gov/ (accessed on 19 July 2025)) under accession nos. PV664587-PV664589, PV672203-PV672205, PV672206-PV672209, PV683347-PV683349, PV683351, and PV700082.

## Supplementary Materials

The following supporting information can be downloaded at https://www.mdpi.com/article/10.3390/microorganisms13081731/s1: Table S1: Sequences of primers and thermal cycling programs utilized in each nested PCR; Table S2: Information on the geographical distribution of AIDS participants; Table S3: Accession nos. and geographical origins of target gene sequences from GenBank used for sequence analyses; Table S4: Detailed information on test-positive participants.

## Abbreviations

The following abbreviations are used in this manuscript:

AIDS	Acquired immunodeficiency syndrome
Accession no.	Accession number
*bg*	β-gardin
BSA	Bovine serum albumin
CD4+	Cluster of differentiation 4 positive
*gp60*	60-kDa glycoprotein gene
*gdh*	Glutamate dehydrogenase
HIV	Human immunodeficiency virus
ITS	Internal transcribed spacer
PBS	Phosphate buffered saline
PCR	Polymerase chain reaction
PLHIV	People living with human immunodeficiency virus
SSU rRNA	Small subunit ribosomal RNA
*tpi*	Triosephosphate isomerase
