# Supplementary material for: Emergence of Cryptosporidium parvum IIc Subtype and Giardia duodenalis Assemblage E in AIDS Patients in Central China: Evidence for Neglected Transmission Dynamics
Source: Microorganisms. 2025 Jul 24;13(8):1731. doi: 10.3390/microorganisms13081731 (PMC12388234; doi:10.3390/microorganisms13081731)
Supplement: Supplementary file 1 [file microorganisms-13-01731-s001.zip › microorganisms-3705017-supplementary.pdf]

Table S1. Sequences of primers and thermal cycling programs utilized in each nested PCR

Species	Target genes	Reaction	Primers	Sequences (5'-3')	Annealing temperature[1] (°C)
Cryptosporidium species	*SSU rRNA*	Primary	18S-Xiao-F1	TTCTAGAGCTAATACATGCG	55
			18S-Xiao-R1	CCCATTTCCTTCGAAACAGGA	
		Secondary	18S-Xiao-F2	GGAAGGGTTGTATTTATTAGATAA AG	55
			18S-Xiao-R2	CTCATAAGGTGCTGAAGGAGTA	
	gp60 (*C. parvum* and *C. hominis*)	Primary	LX0374	TTACTCTCCGTTATAGTCTCC	52
			LX0375	GGAAGGAACGATGTATCTGA	
		Secondary	AL3532	TCCGCTGTATTCTCAGCC	55
			AL3534	GCAGAGGAACCAGCATC	
	gp60 (*C. meleagridis*)	Primary	CRSout115F	GATGAGATTGTCGCTCGTTAT C	60
			CRSout1328R	AACCTGCGGAACCTGTG	
		Secondary	ATGF mod	GAGATTGTCGCTCGTTATCG	58
			GATR2	GATTGCAAAAACGGAAGG	
Enterocytozoon bieneusi	*ITS*	Primary	Eb-ITS-F1	GATGGTCATAGGGATGAAGAGCTT	55
			Eb-ITS-R1	TATGCTTAAGTCCAGGGAG	
		Secondary	Eb-ITS-F2	AGGGATGAAGAGCTTCGGCTCTG	55
			Eb-ITS-R2	AGTGATCCTGTATTAGGGATATT	
Giardia duodenalis	*bg*	Primary	BG-F1	AAGCCCGACGACCTCACCCGCAGTGC	56
			BG-R1	GAGGCCGCCCTGGATCTTCGAGACGAC	
		Secondary	BG-F2	GAACGAGATCGAGGTCCG	58
			BG-R2	CTCGACGAGCTTCGTGTT	
	tpi	Primary	TPI-F1	AAATIATGCCTGCTCGTCG	56
			TPI-R1	CAAACCTTITCCGCAAACC	
		Secondary	TPI-F2	CCCTTCATCGGIGGTAACTT	62

		TPI-R2	GTGGCCACCACICCCGTGCC	
gdh	primary	GDH-F1	TTCCGTRTYCAGTACAACTC	52
		GDH-R1	ACCTCGTTCTGRGTGGCGCA	
	secondary	GDH-F2	ATGACYGAGCTYCAGAGGCACGT	60
		GDH-R2	GTGGCGCARGGCATGATGCA	

[1]The complete thermal cycling program contains: 94 ℃ for 5 min, followed by 35 cycles at 94 ℃ for 30 s, (annealing temperature) for 30 s, 72 ℃ for (60 s / kb) s, and final extention at 72 ℃ for 7 min.

Table S2. Information on the geographical distribution of AIDS participants

Province	City	Residential Area		Number of participants
		Urban	Rural	
Hubei	Wuhan	35	17	52
	Suizhou	2	7	9
	Jingzhou	1	8	9
	Xianning	5	3	8
	Enshi	0	6	6
	Yichang	1	2	3
	Shiyan	0	3	3
	Tianmen	1	2	3
	Qianjiang	0	2	2
	Huangshi	0	1	1
	Jingmen	0	1	1
	Ezhou	1	0	1
	Xiantao	1	3	4
	Jingmen	0	1	1
Jiangxi	Jiujiang	1	0	1
Sichuan	Chengdu	0	1	1
Henan	Xinyang	1	2	3
No information				5
Total				150

Table S3. Accession nos. and geographical origins of target gene sequences from GenBank used for ssequence analyses

Species (gene locus)	Subtypes or assemblages	Geographical location	Host	Accession nos.
Cryptosporidium hominis (*gp60*)	IfA12G1	Mozambique	human	MW480836
				AY166810
		United Kingdom	human	EU161655
				HQ149036
		Netherland	human	MH796380
		Australia	human	GU933448
				GU933447
				GU810910
				MK165982
		Southern Ireland	human	MT053131
Cryptosporidium parvum (*gp60*)	IIcA5G3	South Africa	human	AF440621
				AF440636
		Jamaica	human	EU141721
		Mozambique	human	AF440631
				KX579757
Cryptosporidium meleagridis (*gp60*)	IIIbA22G1R1c	India	human	KJ210607
		China	human	KY575457
		China	chicken	MG969391
Giardia duodenalis (tpi)	Assemblage B	Slovenia	human	LN626348
	Assemblage A	China	human	OR453274
				GU564275
			black-boned goat	MK327162
			cattle	OL694043

	cattle	OP189550
	sheep	MN833280
	yak	MH230890
	tortoise	KR051229
	dunkey	OR497204
	chipmunk	MF671916
	Trachypithecus francoisi	KJ888991
	musk deer	MF497412
	ferret	PP578225
	persian fallow deer	KR051228
	bactrian camel	MT319053
Spain	*Lynx pardinus*	OR921176.1
	sheep	JF792423
Brazil	human	MZ822181
	dog	KT728527
Peru	*Vicugna pacos*	JX845450
India	human	PP584162
Iran	human	OP312605
Belgium	foal	KM926531
Malaysia	human	HQ836660
	pig	PQ834760
Greece	foal	KM926546
	sheep	KM926546
Japan	cat	AB569393
	Mustela putorius furo	AB509384
Australia	human	EF688031

			lamb	GQ444447
			Bubalus bubalis	KF019193
		Norway	*Rangifer tarandus tarandus*	MH155690
		USA	*Odocoileus virginianus*	AY302562
			sheep	JQ688289
Giardia duodenalis (bg)	Assemblage E	Norway	sheep	GQ337972
		China	Tan sheep	MK610389
			cattle	MK252653
				KY769091
				KT369772
				OM001830
				KC960638
				ON773555
			horse	MN174850
			sheep	MN833266
			Tibetan sheep	KY633467
			Tibetan sheep	MK573342
		Bangladesh	cattle	KJ188071
				KJ188068
				KJ188069
				KJ188084
		India	cattle	GQ290390
		South Korea	cattle	ON677358
				ON677353
				ON677355

	USA	cattle	OM653969
Assemblage B	Japan	human	AB618785
	China	hylobatidae	KY696833
			KY696834
	Canada	human	KP687755
			KM190794
	Poland	human	EU626199
			FJ009207
			FJ009209
	New Zealand	human	EU274389
	Italy	human	EU637581
			EU637579
	Malaysia	human	KT124854
	USA	human	KP780910
	Romania	human	PP374765
	Sweden		HM165214
	Iran	human	LC745020
			LC184018
			LC183972
	Kenya	human	LC436574
	Brazil		KU504724
			MG807899
			KU504724
	Norway	human	DQ090528
			DQ090524

		India	human	JF918500
				GQ345010
		Egypt	human	HM171689
		Nigeria	human	OP947121
		Kenya	human	LC436571
		Dominican Republic	human	ON807278
Giardia duodenalis (*gdh*)	Assemblage B	Brazil	human	EF507682
		China	golden monkey	MK952603
			Japanese macaque	KF679730
			ring-tailed lemur	MK952599
			hylobatidae	KY696801
				KY696802
				KY696804
				KY696794
				KY696795
				KY696798
				KY696799
			rabbit	OM001860
			rabbit	KC960645

Table S4. Detailed information on test-positive participants

Details	Participants							
	No. 1	No. 2	No. 3	No. 4	No. 5	No. 6	No. 7	No. 8
Species/assemblage infected	*G. duodenalis* assemblage B	*G. duodenalis* assemblage B	*G. duodenalis* assemblage B	*G. duodenalis* assemblage E	*G. duodenalis* assemblage A	*C. hominis*	*C. meleagridis*	*C. parvum*
Age	62	56	35	47	43	35	54	37
Gender	male	male	male	male	male	male	female	male
Residential area	rural area	rural area	rural area	rural area	rural area	rural area	rural area	rural area
Exposure or contact with animals	denied	denied	denied	denied	denied	denied	denied	denied
Drinked raw water	denied	denied	denied	Yes	denied	Yes	Yes	Yes
Ate raw vegetables	denied	denied	denied	denied	denied	Yes	denied	denied
Receiving ART	Yes	Yes	No	No	Yes	No	No	No
